# Genome-Wide *De Novo* Prediction of *Cis*-Regulatory Binding Sites in *Mycobacterium tuberculosis* H37Rv

**DOI:** 10.1371/journal.pone.0148965

**Published:** 2016-02-17

**Authors:** Wei Wu, Xian Sun, Yun Gao, Jun Jiang, Zhenling Cui, Baoxue Ge, Hai Wu, Lu Zhang, Yao Li

**Affiliations:** 1 State Key Lab of Genetic Engineering, Shanghai Engineering Research Center of Industrial Microorganisms, College of Life Sciences, Fudan University, Shanghai, PR China; 2 Shanghai Key Laboratory of Tuberculosis, Shanghai Pulmonary Hospital, Medical School, Tongji University, Shanghai, China; University of Texas at San Antonio, UNITED STATES

## Abstract

The transcription regulatory system of *Mycobacterium tuberculosis* (*M*. *tb*) remains incompletely understood. In this study, we have applied the eGLECLUBS algorithm to a group of related prokaryotic genomes for *de novo* genome-wide prediction of *cis*-regulatory binding sites (CRBSs) in *M*. *tb* H37Rv. The top 250 clusters from our prediction recovered 83.3% (50/60) of all known CRBSs in extracted inter-operonic sequences of this strain. We further demonstrated that the integration of our prediction results with the ChIP-Seq datasets is very effective in identifying true binding sites of TFs. Using electrophoretic mobility shift assays and real-time RT-PCR, we experimentally verified our prediction of CRBSs for Rv0081, an important transcription factor thought to be involved in regulation of *M*. *tb* under hypoxia.

## Introduction

*Mycobacterium tuberculosis* (*M*. *tb*), the causative agent of tuberculosis, is one of the leading causes of death and morbidity worldwide. Regulation of biological functions of the pathogen is largely governed by interactions between transcription factors (TFs) and their corresponding *cis*-regulatory binding sites (CRBSs) encoded in the intergenic regulatory sequences in genomes. Therefore, identification of CRBSs for each TF in the genome will allow us to better understand the transcription regulatory networks of this important pathogen.

Over the past several years, the number of genome sequences of *Mycobacterium* species has increased tremendously, making it possible to employ computational methods to predict CRBSs at the genomic level. Midha *et al*. firstly used phylogenetic footprinting technique [1] for *de novo* prediction of all CRBSs in *M*. *tb* genome [2]. However, they used a single motif finding tool (MEME) for the analysis, which would lead to an incomplete result without other complementary tools. Also, they assumed all predicted motifs generated by MEME were true motifs, therefore, they did not differentiate authentic motifs from spurious ones. Hence, it is necessary to predict the CRBSs of *M*. *tb* with higher precision and coverage, using improved algorithms.

Zhang *et al*. recently designed the algorithm ‘GLECLUBS’ (GLobal Ensemble CLUsters of Binding Sites) for genome-wide *de novo* predication of *cis*-regulatory binding sites in prokaryotes [3], which is based on comparative genomics and takes advantage of the complementary nature of some of the well-regarded algorithms. Zhang *et al*. showed that the algorithm can predict 81% of known binding sites belonging to 94% of known *cis*-regulatory motifs in the *E*. *coli* K12 genome, while achieving high prediction specificity. It has also achieved similar prediction accuracy in the *B*. *subtilis* genome, suggesting that the GLECULBS algorithm is robust and can be applied to other prokaryotic genomes [3]. More recently, they developed a new algorithm based on GLECLUBS called extended GLECLUBS (eGLECLUBS) for simultaneous prediction of CRBSs in a group of related prokaryotic genomes. This algorithm has achieved the same level of accuracy and robustness as its predecessor GLECLUBS, but can work on dozens of genomes at the same time [4].

In this study, we employed eGLECLUBS for genome-wide *de novo* prediction of CRBSs in *M*.*tb* H37Rv, a well-studied laboratory virulent strain of *M*. *tb*. We successfully recovered 83.3% (50/60) of known CRBSs in extracted inter-operonic sequences of *M*. *tb* H37Rv. Furthermore, we integrated our prediction results with the ChIP-Seq datasets of 81 TFs that have been recently released from TBDB [5] and mapped each of these TFs to its corresponding binding sites in the *M*. *tb* genome. We experimentally verified our prediction of Rv0081, an important transcription factor thought to be involved in regulation of hypoxia.

## Materials and Methods

### Genomic materials, operons files and ChIP-Seq datasets

The genomic materials of all *actinomycetales* (158 available), including the sequences of gene, protein, genome and annotation files, were downloaded from NCBI ftp (ftp://ftp.ncbi.nih.gov/genomes/Bacteria) on November 13th, 2013. TFs of *M*. *tb* were predicted by DBD database [6]. Operons files of 35 selected target genomes were predicted by DOOR database [7]. The ChIP-Seq datasets on 81 TFs of *M*. *tb* H37Rv were retrieved from TBDB [5].

### Selection of target genomes

The algorithm is based on comparative genomics and the selection of proper reference genomes for *M*. *tb* H37Rv is critical for the accurate prediction of CRBSs. We firstly chose 158 candidate genomes (*M*. *tb* H37Rv included) of the order *actinomycetales* (see S1 Fig). After the analysis of transcriptional regulatory similarities, 35 genomes belonging to the suborder *corynebacterineae* were selected as the target genomes. Each target genome has at least 50% TF orthologs in *M*. *tb* H37Rv (see S2 Fig).

### Prediction of CRBSs using eGLECLUBS

We predicted the H37Rv-specific CRBSs by following the steps of eGLECLUBS. Briefly, these steps included identification of orthologous relationships between operons, prediction of clusters of operons with orthologous relationships (COOR), prediction of motifs for each COOR, construction of motif similarity graphs and refine and rank the clusters of *M*. *tb* H37Rv. More detailed methods are described in S1 File.

### Integration of CRBSs prediction results with the ChIP-Seq dataset

ChIP-Seq datasets released from TBDB consist of binding sequences of 81 TFs of the *M*. *tb* H37Rv. For the convenience of discussion, in this paper, we call such a binding sequence in ChIP-Seq datasets CBS for short. For each of the 81 TFs, we first extracted corresponding CBSs above the 80^th^ percentile for peak height from the database, and then mapped the predicted binding sites (PBS) of the top 250 clusters produced by eGLECLUBS to each CBS. There may be a certain number of PBSs present in each CBS. All PBSs mapped to the CBSs of the TF were classified by the clusters already. Finally, we used the hypergeometric test with multiple test correction in R software to discover the overrepresented clusters. A *p* value of 0.05 was considered to have statistical significance. Theoretically, the most significant cluster should be the binding motif of the TF. Motif candidates of 65 TFs in the datasets were successfully discovered in the analysis.

### Bacterial growth conditions, production of recombinant protein Rv0081a and RNA extraction

*M*. *tb* H37Rv strain was grown in Middle-brook 7H9 broth medium. Detailed methods about growth conditions, plasmid construction, recombinant protein purification and RNA extraction are described in S1 File.

### Real-time reverse transcription PCR (RT-PCR)

Genomic DNA was removed before reverse transcription using the PrimerScript RT reagent kit (TAKARA) was carried out. Reverse transcription was performed with random primers. Quantitative PCR was performed with SYBR green mix (CW-bio). Primers for RT-PCR are described in S1 File.

### Electrophoretic mobility shift assays (EMSA)

10 nucleotides from predicted binding sites of *M*. *tb* H37Rv are listed in Table 1. Each nucleotide with its reverse complementary sequence were annealed at 85°C for 5 min and cooled to room temperature to generate 24bp double-stranded DNA. These double-stranded DNA were then incubated with purified Rv0081 protein in a reaction buffer [20mM KCl, 5% glycerol, 25mM Tri-HCl, 6mM MgCl_2_, 0.5 mM EDTA, 0.5μg of poly (dI-dC), pH = 8.0] for 30 min at room temperature. Following incubation, binding reaction mixtures were loaded onto 6.5% nondenaturing polyacrylamide gels and electrophoresed at 70V for about 1h at 4°C. Gels were dried in nucleic acid dye (Gelsafe, YuanPingHao Bio) and then photoed.

**Table 1:** Overrepresented clusters of each TF with known binding sites.

TF	Known binding sites	Overrepresented clusters		
		Rank	PBS	P-value
Rv1033c	KBS_14(**PBS_249**–23), KBS_15(PBS_9–84)	1	**PBS_249**	3.12E-03
		2	PBS_221	3.12E-03
Rv1846c	KBS_19(**PBS_195**–15)	1	PBS_28	3.40E-03
		2	PBS_217	3.40E-03
		3	**PBS_195**	5.80E-03
Rv1994c	KBS_21(**PBS_205**–13)	1	**PBS_205**	6.94E-04
		2	PBS_27	6.94E-04
		3	PBS_158	6.94E-04
Rv2359	KBS_23(**PBS_76**–4), KBS_24(**PBS_76**–5),	1	**PBS_76**	4.42E-21
	KBS_25(**PBS_76**–3), KBS_26(**PBS_76**–6),	2	PBS_146	1.63E-03
	KBS_27(**PBS_76**–10), KBS_28(**PBS_76**–1)	3	PBS_209	5.80E-03
Rv2506	KBS_31(**PBS_65**–17)	1	**PBS_65**	4.87E-04
		2	PBS_171	4.87E-04
		3	PBS_156	4.92E-04
Rv3066	KBS_46(**PBS_221**–6)	1	PBS_250	1.94E-04
		2	PBS_142	2.05E-04
		3	PBS_138	9.99E-04
		4	PBS_40	3.50E-03
		5	**PBS_221**	4.07E-03
Rv3133c	KBS_47(**PBS_51**–6), KBS_48(**PBS_149**–9)	1	**PBS_51**	2.48E-40
		2	**PBS_149**	5.53E-03
		3	PBS_219	5.53E-03
Rv3574	KBS_50(**PBS_11**–21), KBS_51(**PBS_11**–2),	1	**PBS_11**	3.17E-53
	KBS_52(**PBS_11**–4), KBS_53(**PBS_11**–7),	2	PBS_182	4.57E-02
	KBS_54(**PBS_11**–13), KBS_55(**PBS_11**–12)	3	PBS_19	4.58E-02
	KBS_56(**PBS_11**–14), KBS_57(**PBS_11**–19),			
	KBS_58(**PBS_11**–18), KBS_59(**PBS_11**–1),			
	KBS_60(**PBS_11**–27), KBS_61(**PBS_11**–6)			
Rv3855	KBS_67(**PBS_46**–72)	1	PBS_152	4.12E-04
		2	**PBS_46**	6.28E-04
		3	PBS_110	6.28E-04

Abbreviation: KBS, known binding sites; PBS, predicted binding sites. PBSs corresponding to KBSs are shown in parentheses. For each TF, overrepresented predicted clusters containing KBSs are indicated in bold. *P* values were calculated by the hypergeometric test with multiple test correction.

## Results

### Prediction of CRBSs in *M*. *tb* H37Rv using eGLECLUBS

**Fig 1:**
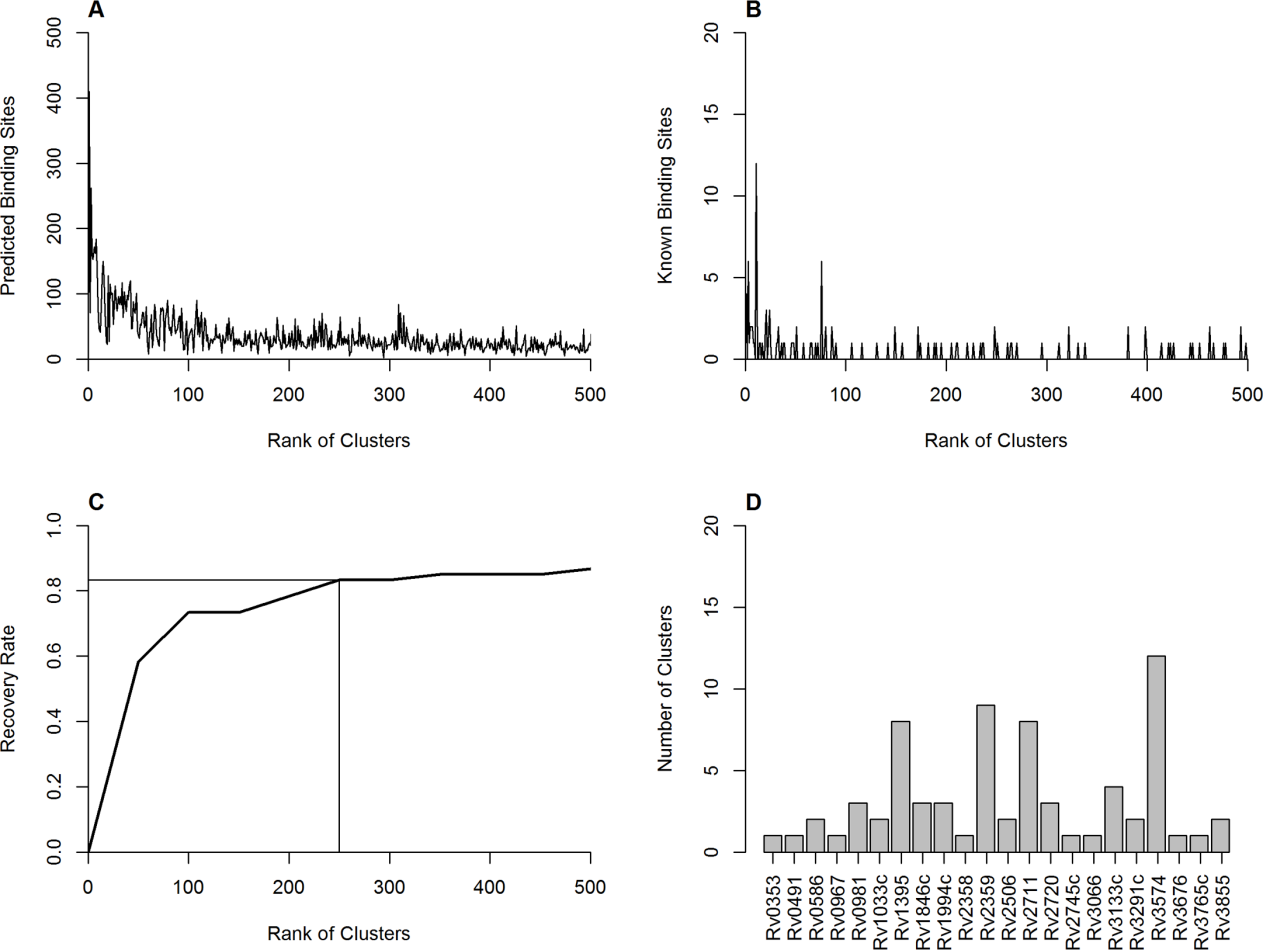
Evaluation of the top-ranked clusters. (A) The number of predicted binding sites in the top 500 clusters. (B) The number of known binding sites recovered by the top 500 clusters. (C) Cumulative recovery rate of the known binding sites in the input motifs by the top-ranked cluster, computed as the ratio of the number of cumulative known binding sites recovered in top-ranked clusters to the number of known binding sites in the set of input motifs. (D) The number of predicted clusters of the top 250 clusters for the known TFs of *M*. *tb* H37Rv.

The output of the *de novo* prediction of CRBSs in *M*. *tb* H37Rv is a ranked list of 5364 clusters (see S1 Table). Each cluster consists of several CRBSs from different inter-operonic regions. The CRBSs in the same cluster are presumably recognized by a certain TF. The top-ranked clusters are thought to have higher quality and the CRBSs in these clusters tend to be true binding sites. Also, the top-ranked clusters generally contain more PBSs than those ranked lower (Fig 1A).

To assess the performance of our prediction, we compared the prediction results with experimentally verified CRBSs. We consider a predicted sequence as a true binding site of a particular TF if this sequence has been experimentally confirmed by electrophoretic mobility shift assays (EMSA). After reviewing the publicly available literatures on EMSA-confirmed binding sites of *M*. *tb* H37Rv, we have assembled a total of 67 known binding sites, or KBSs for short, for 25 different TFs (see S2 Table). Seven KBSs were located in intergenic regions but not in inter-operonic regions by our operon prediction.

Next, we analyzed the distribution of these KBSs in the predicted clusters. Most of these KBSs are within the top-ranked clusters (Fig 1B). We then calculated the recovery rate of the 60 KBSs that are located at the correctly extracted inter-operonic regions. As shown in Fig 1C, the recovery rate of these KBSs increases rapidly within the top 100 clusters, and increases more slowly between 100 to 250 clusters. The recovery rate reaches saturation after the top 250 clusters, recovering 83.3% (50/60) of the KBSs. Based on these results, we selected the top 250 clusters as the potential *cis*-regulatory binding motifs for the estimated 200 transcriptional regulators in the *M*. *tb* H37Rv genome. S3 Fig displays the top 10 clusters/motifs of our prediction. All of them have palindromic, or tandem repeat structures, suggesting that they are likely to be true binding sites.

We mapped the recovered KBSs with the PBSs of the top 250 clusters. 119 PBSs were mapped to 50 KBSs. Since the lengths of KBSs verified by EMSA experiments are often longer than that of PBSs, it is obvious that several PBSs map to a single KBS (see S3 Table). For the 25 TFs with known binding sites, we analyzed their KBSs in the top 250 predicted clusters, respectively. There are 22 TFs having KBSs in the top 250 clusters, indicating a high coverage for known TFs in H37Rv (Fig 1D). Most of them (21 of 22) have fewer than 10 clusters.

### Integration of CRBS prediction with ChIP-Seq datasets to map TF binding sites

Knowing the CRBSs in *M*. *tb* on a genome-wide scale is only the beginning to understand the complex transcriptional regulatory network of this bacterium since we do not know which TF binds to which CRBSs. To map each TF to its corresponding binding sites, we integrated the ChIP-Seq datasets of 81 TFs, which were retrieved from TBDB, with our CRBSs prediction (see ‘Methods‘). 16 TFs in the datasets were not included in the final analysis due to the very limited number of CBSs. Final results consist of the remaining 65 TFs and the overrepresented clusters and binding sites for each TF (see S4 Table). We analyzed the distribution of these clusters in S4 Table. For all the clusters of 65 TFs, the median rank is 98 and most of the clusters ranked high among top 250 clusters.

Then, we used the KBSs to evaluate the accuracy of the overrepresentation analysis. Among the 65 TFs with available ChIP-Seq data, 11 of them have KBSs that were previously confirmed by experiments and described in literatures, which are Rv0465c, Rv0967, Rv1033c, Rv1846c, Rv1994c, Rv2359, Rv2506, Rv3066, Rv3133c, Rv3574 and Rv3855 [8–17]. We tested if we can successfully identify the KBSs in overrepresented clusters of these TFs. As shown in Table 1, our search for overrepresented clusters of each TF successfully identified the KBSs for nine TFs. For six TFs (Rv1033c, Rv1994c, Rv2359, Rv2506, Rv3133c and Rv3574), the top-ranked clusters from our calculation match the KBSs. For Rv3133c, the KBS matches Cluster 51 and Cluster 149, which is ranked as the first and second possible binding motif respectively.

### Experimental verifications for predicted binding sites of Rv0081 using EMSA experiments

**Fig 2:**
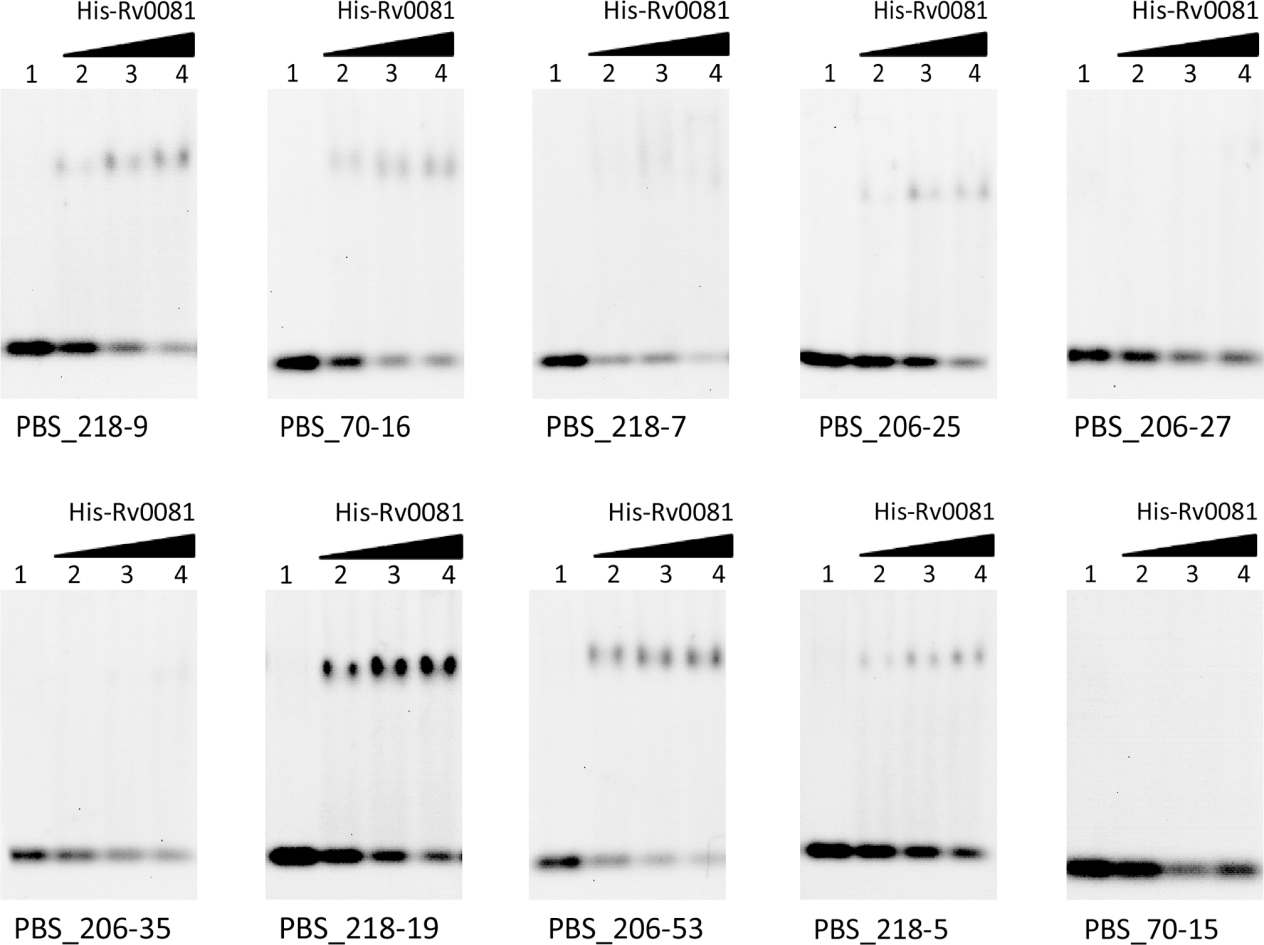
Electrophoretic mobility shift assay for Rv0081. For each PBS, the DNA (10 mM) was incubated with increasing concentrations (0, 1nM, 1.7nM and 2.3nM) of the purified Rv0081 protein (lanes 1–4, respectively).

After integrating our prediction with the ChiP-Seq data, we narrowed down the potential binding sites recognized by each TF. The integration facilitates our attempts to identify true binding sites for TFs regulating important biological functions. Rv0081 is a regulatory hub and has a broad regulatory role in the initial hypoxic response [18]. The binding motifs of this TF were PBS_206, PBS_218 and PBS_70 according to our prediction (see S4 Table). To verify our prediction, we selected 10 PBSs belonging to the three motifs as candidate binding sites according to the descending percentiles in ChIP-Seq data to carry out EMSA experiments (Table 2). Most of the PBSs selected (PBS_218–9, PBS_70–16, PBS_218–7, PBS_206–25, PBS_218–19, PBS_206–53, PBS_218–5) were shifted by the protein in the EMSA experiment, demonstrating interactions between Rv0081 and the selected PBSs under in vitro conditions (Fig 2).

**Table 2:** Predicted binding sites of Rv0081 selected for experimental verifications.

ID	Name	Sequence	Target	P value
1	PBS_218–9	GAAAGTTC	Rv2778c	4.44E-02
2	PBS_70–16	GATGCAACGTGCAT	Rv3619c	1.26E-03
3	PBS_218–7	GAGAATTT	Rv1057	4.44E-02
4	PBS_206–25	TAGACGCTAC	Rv0505c	1.26E-03
5	PBS_206–27	TGGGAACAAG	Rv2329c	1.26E-03
6	PBS_206–35	CGAGCCCAAT	Rv2329c	1.26E-03
7	PBS_218–19	AAAACTTC	Rv0002, Rv0003, Rv0004	4.44E-02
8	PBS_206–53	AGTTTGAAAT	Rv2145c	1.26E-03
9	PBS_218–5	GAGAATTC	Rv1503c, Rv1504c	4.44E-02
10	PBS_70–15	GGTGTAGTTCGCAC	Rv2699c	1.26E-03

P values were calculated by the hypergeometric test with multiple test correction.

### Experimental verifications for potential targets of Rv0081 using real-time RT-PCT after up-regulation of Rv0081

**Fig 3:**
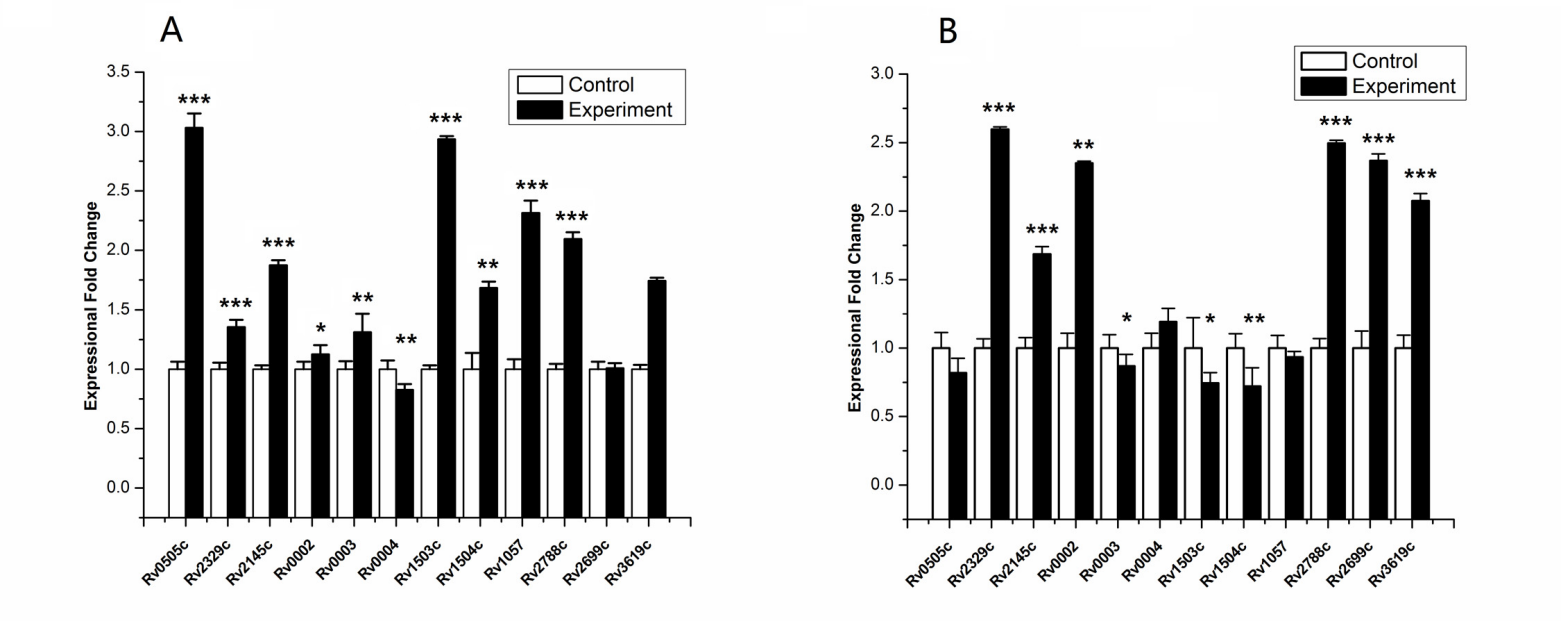
Confirmation of Expression fold changes of potential target proteins of Rv0081 by real-time RT-PCR. Expression fold change of down-stream genes of PBSs mentioned previously after upregulated Rv0081 in *M*. *tb* H37Rv stain. RNA samples were extracted at exponential growth phase (14days) under two conditions. Results are shown as average fold-change (up-regulated plamid/empty plasmid as control) values of two conditions, hypoxic conditions (A) and normal conditions (B). (p<0.05, *; 0.001<p<0.05, **; p<0.001, ***).

For these PBSs, we also analyzed the potential targets in our previous prediction. Over expression of Rv0081 in *M*. *tb* H37Rv was predicted to affect the expression levels of these targets. Since Rv0081 was reported to mediate the initial response to hypoxia, we analyzed the expression levels of the targets under both hypoxic and normal conditions and found all targets were up-regulated under at least one condition (Fig 3).

## Discussion

In this study, we employed the eGLECLUBS algorithm to predict CRBSs in the genome of *M*. *tb* and compiled the top 250 clusters that likely represent the majority of binding motifs of the estimated 200 TFs in *M*. *tb*. Our CRBSs prediction recovered 83.3% of known binding sites in the top 250 clusters. We further demonstrated that the availability of the whole genome CRBSs, in combination with ChIP-Seq data, is very effective for identifying true binding sites of TFs.

Midha *et al*. (2012) recently identified regulatory regions in *Mycobacterium* species, and developed a database, MycoRRdb. They reported that 64.1% (116/181) retrieved known CRBSs from *Mycobacterium* species were mapped in MycoRRdb [2]. In our eGLECLUBS prediction, we have mapped 83.3% known CRBSs of *M*. *tb* H37Rv in the top 250 clusters. The higher prediction coverage of eGLECLUBS may be related to three factors: genome selection, motif prediction and motif clustering. Firstly, we selected the target genomes based on both phylogenetic relationships and similarities of transcriptional regulatory network. Also, since mycobacterial species grouped in the *M*. *tb* complex (e.g., *M*. *tb*, *M*. *bovis* including BCG, *M*. *africanum*) are characterized by 99.9% similarity at the nucleotide level and identical 16S rRNA sequences [19], we excluded strains that are very closely related to avoid overrepresentation in the dataset. Secondly, Midha *et al*. used only one motif finding tool (MEME) to uncover motifs, whereas we included, in addition to MEME, four other complementary motif-finding algorithms (BioProspector, CUBIC, MDscan and MotifSampler) in our analyses. These five algorithms exhibit complementary prediction effect and collectively lead to more accurate prediction of true binding sites [3]. Finally, Midha *et al*. assumed all predicted motifs generated by MEME were true motifs and could not differentiate authentic motifs from spurious ones. In our prediction, motifs predicted by all five algorithms were differentiated and clustered into new motifs, which have higher reliability.

The integration of our PBSs to ChIP-Seq data also demonstrates high accuracy of our CRBS prediction. For each TF in TBDB, several clusters were effectively discovered as motif candidates and the most significant one often corresponds to known binding sites of the TF. The successful integration to ChIP-Seq data also suggests that our results can be integrated with ChIP-chip and PBM data.

In theory, binding events should occur in every fragment pulled down by ChIP experiments. However, we found that the coverage of the most significant cluster for each TF is significantly lower than the expected 100%. One reason is that TFs do not always act in isolation, instead may operate in combination with other factors to regulate a particular gene. In such instances, DNA fragments may be pulled down by cofactors of the TF. Also, some DNA fragments identified by the ChIP-Seq experiments may have no natural affinity for the TF and therefore are false positives. It has been estimated that up to 30% of binding sites identified by ChIP-Seq in eukaryotes may be false positives [20]. For these reasons, we mapped the KBSs to ChIP-Seq data to see characteristics of true binding sites. 30 of 67 KBSs were mapped to CBSs of 11 TFs and they are all above the 80^th^ percentile for peak height (data not shown). Thus, we chose the 80^th^ percentile as a cutoff for the overrepresentation analysis.

Our analysis of the 65 TFs with available ChIP-Seq data revealed multiple significant clusters for each TF (see S4 Table). There may be at least two explanations for the results: (1) as mentioned before, some CRBSs of the same motif may be split into different clusters due to low motif similarity. For example, Cluster 51 and Cluster 149 actually represent the same binding motif for Rv3133c (Table 1). This division of the same binding motif into several different clusters may also lower the coverage. (2) Some TFs have more than one true binding motifs. It has been recently suggested that many TFs can recognize multiple distinct sequence motifs and some of which may constitute ‘weak’ binding sites [21]. This has been demonstrated for eukaryotic transcription factors and suggested for *M*. *tb* by analyzing the ChIP-Seq dataset from TBDB [22]. Consistently, our analysis revealed multiple clusters and it remains to be determined if these additional clusters represent true binding sites with differential affinity.

In addition to providing a genome-wide prediction of CRBSs with higher coverage, our work also offers a very reliable algorithm for identifying true binding sites for a specific TF. The success of our approach has been demonstrated in the case of Rv0081. Seven of the ten predicted PBSs for Rv0081 exhibited binding activity with purified Rv0081 protein in EMSA experiments, demonstrating a high prediction accuracy of our results. The remaining three PBSs did not show Rv0081 binding activity in the EMSA assays. These may be false positives of our prediction. On the other hand, since in vitro assays (EMSA) do not fully represent the situation in vivo, the three PBSs may still be true binding sites of Rv0081 in vivo. The potential targets of the PBSs were also up-regulated in hypoxic or normal conditions when Rv0081 was over-expressed in *M*.*tb* H37Rv strain. Since Rv0081 is a regulatory hub and has a broad regulatory role, we speculate that many other PBSs in the three predicted clusters (PBS_206, PBS_218 and PBS_70) may also be true binding sites.

Our results can also serve as a starting point to address some of the biological function of uncharacterized TFs. For example, Rv3574 is involved in lipid degradation of *M*. *tb* [23] and its binding sites were successfully recovered by our analysis and matched cluster 11. Interestingly, Rv0681, a TF of unknown function, also places cluster 11 as the top-ranked cluster and therefore likely its true binding motif (data not shown). Both proteins belong to the TetR transcriptional regulator family. It will be interesting to see if Rv0681 performs a similar function as Rv3574 and if so, how *M*. *tb* coordinates these two TFs.

## Supporting Information

S1 Fig Tree for the selection of reference genomes for the target genome *M*. *tb* H37Rv. We constructed this tree based on the Hamming distance between the TF distribution vectors of each pair of genomes. The number in each brace is the number of TFs shared with *M*. *tb* H37Rv. The 35 genomes labeled with a solid dot are selected as the reference genomes that belong to the monophyletic clades rooted at the star. Some highly similar genomes in the clades are not included to avoid overrepresentation in the reference genomes. Each reference genome has at least 50% TF orthologs sharing with the *M*. *tb* H37Rv genome.
(TIF)

S2 Fig Tree for the 35 target genomes. We constructed this tree based on the Hamming distance between the TF distribution vectors of each pair of genomes. Phylogenetic tree was constructed using the Neighbor-Joining method. The number in each brace is the number of TF orthologs sharing with *M*. *tb* H37Rv.
(TIF)

S3 Fig Motif structures of the top 10 predicted clusters/motifs of *M*. *tb* genome. The logo represents the best motif identified by MEME in each cluster.
(TIF)

S1 File Supplementary materials. (DOC)

S1 Table The output of the prediction of the cis-regulatory binding sites. (XLSX)

S2 Table The 67 known binding sites from literatures. (XLSX)

S3 Table Recovered known binding sites and their predicted binding sites. (XLSX)

S4 Table Overrepresented clusters and binding sites for each TF of 65 TFs in TBDB. (XLSX)
