# Supplementary figures and images for: Genome-Wide De Novo Prediction of Cis-Regulatory Binding Sites in Mycobacterium tuberculosis H37Rv
Source: PLoS One. 2016 Feb 17;11(2):e0148965. doi: 10.1371/journal.pone.0148965 (PMC4757040; doi:10.1371/journal.pone.0148965)

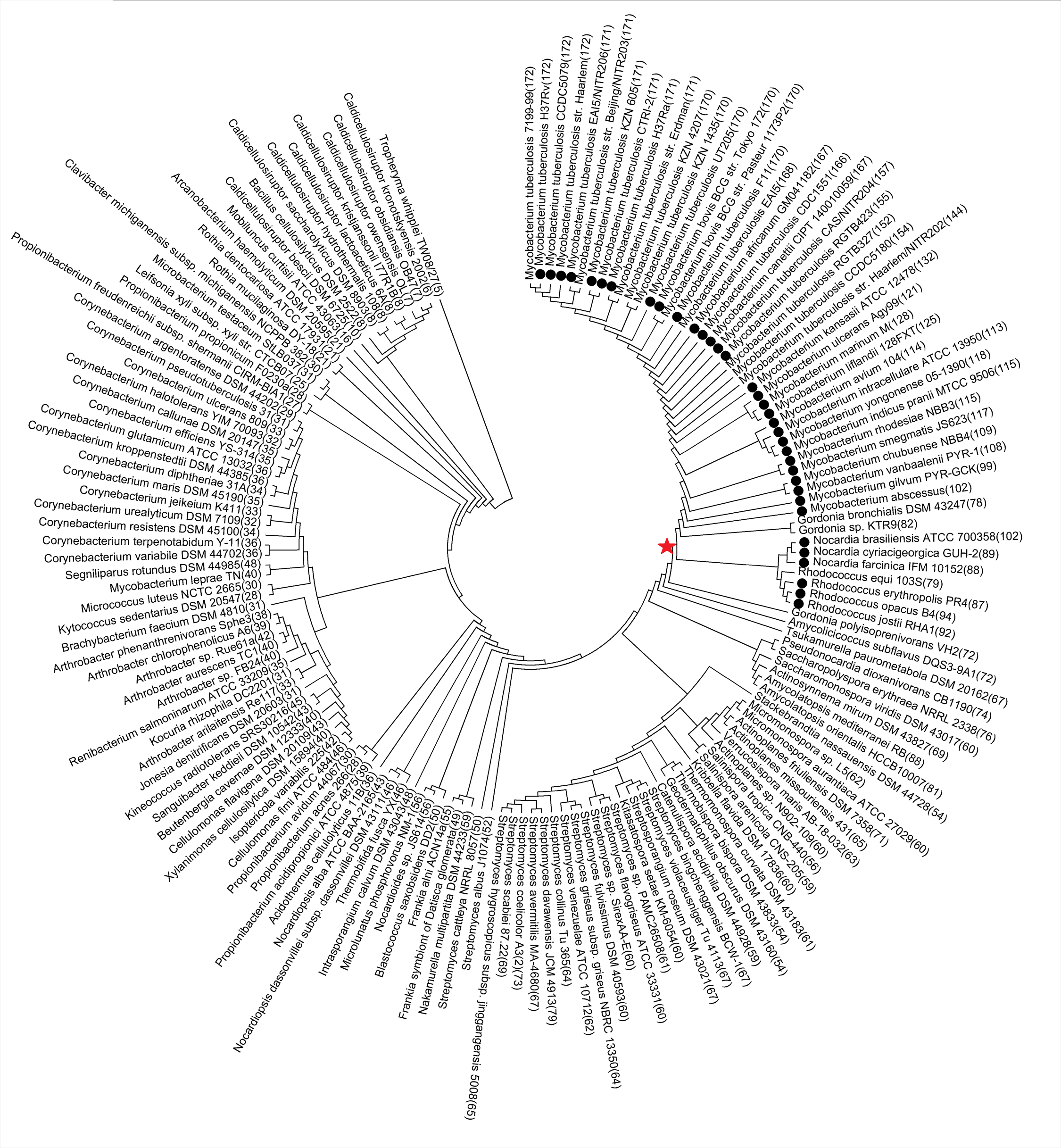

Supplement: S1 Fig — We constructed this tree based on the Hamming distance between the TF distribution vectors of each pair of genomes. The number in each brace is the number of TFs shared with M. tb H37Rv. The 35 genomes labeled with a solid dot are selected as the reference genomes that belong to the monophyletic clades rooted at the star. Some highly similar genomes in the clades are not included to avoid overrepresentation in the reference genomes. Each reference genome has at least 50% TF orthologs sharing with the M. tb H37Rv genome. (TIF) [file pone.0148965.s001.tif]

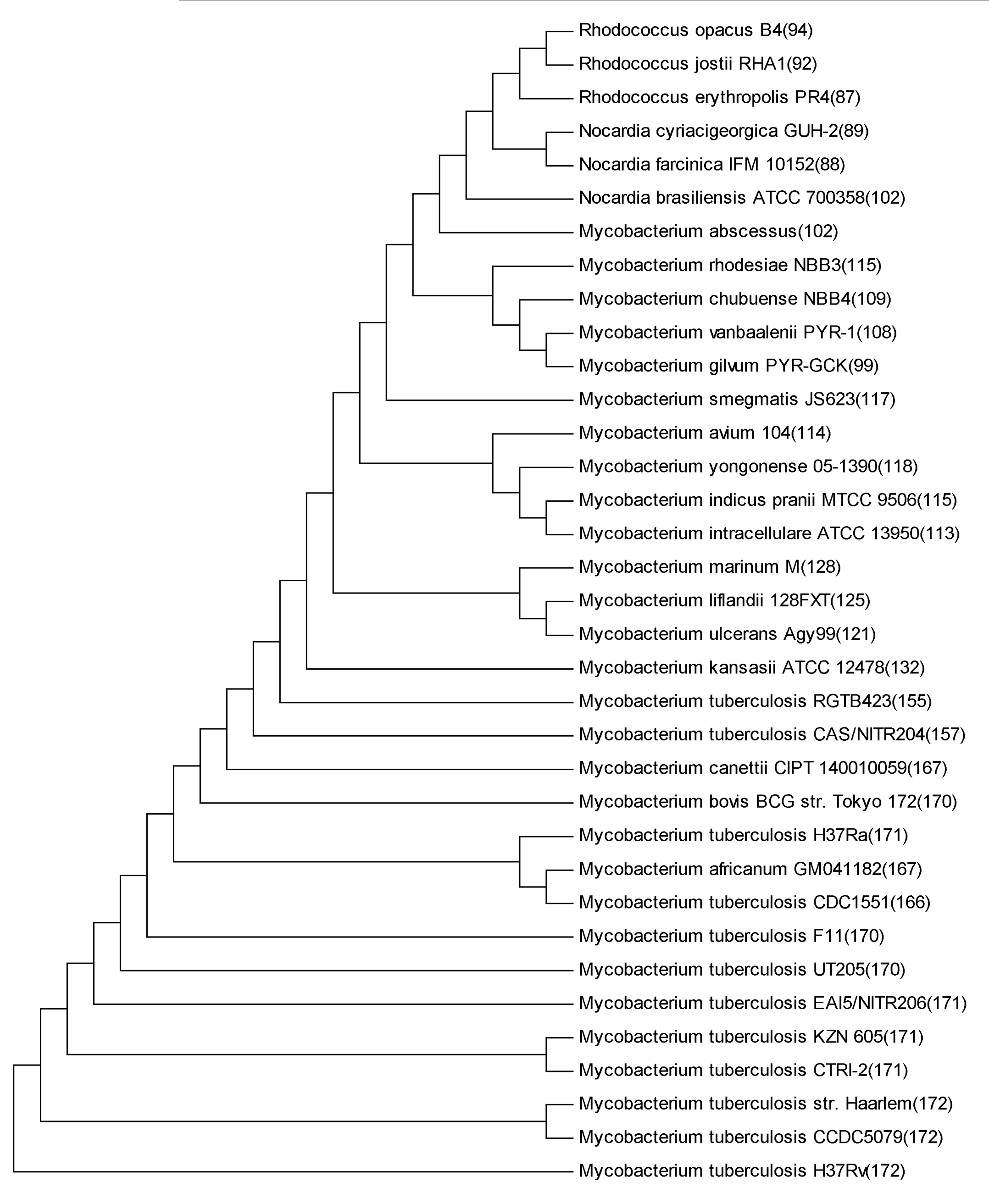

Supplement: S2 Fig — We constructed this tree based on the Hamming distance between the TF distribution vectors of each pair of genomes. Phylogenetic tree was constructed using the Neighbor-Joining method. The number in each brace is the number of TF orthologs sharing with M. tb H37Rv. (TIF) [file pone.0148965.s002.tif]

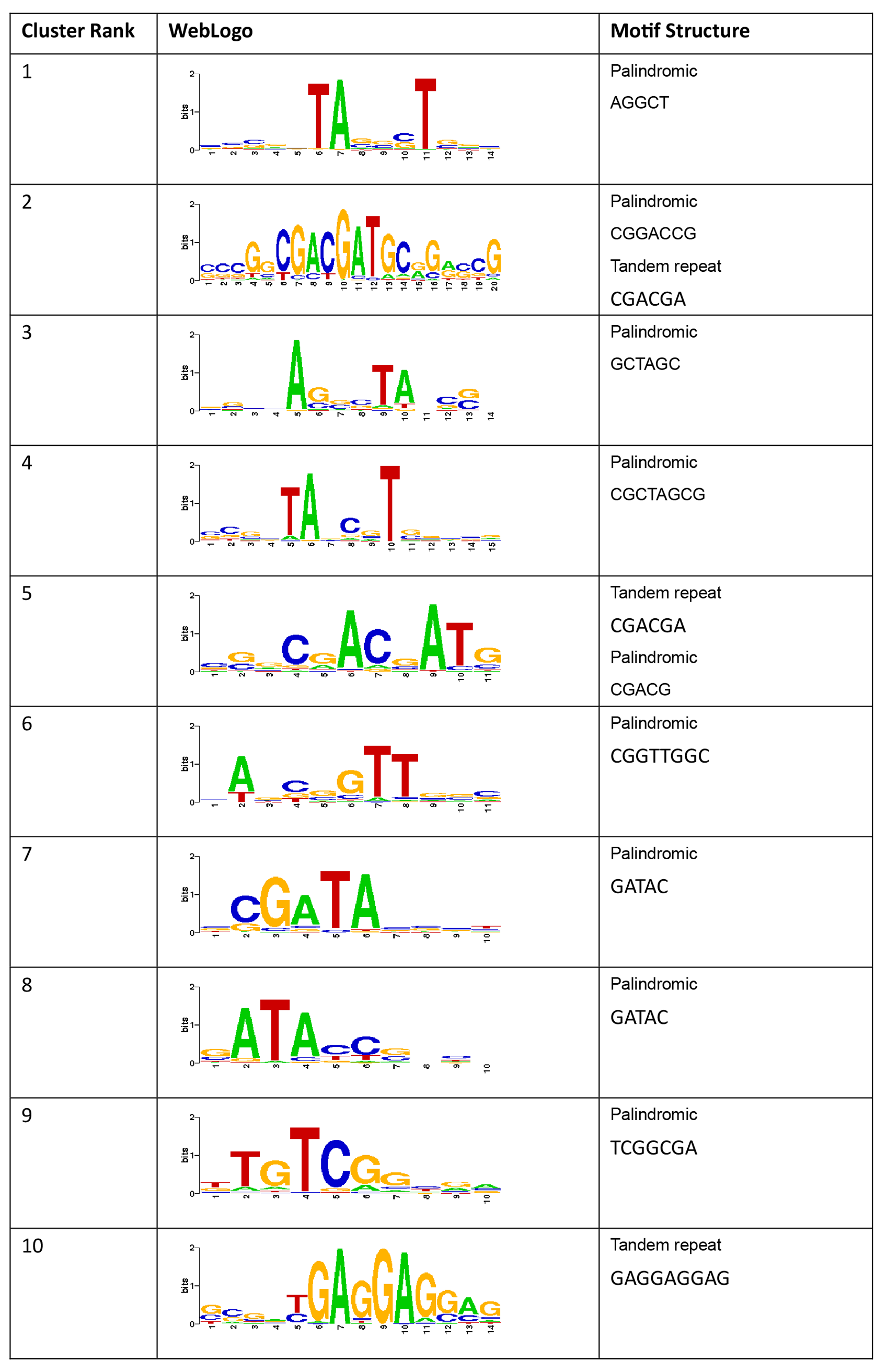

Supplement: S3 Fig — The logo represents the best motif identified by MEME in each cluster. (TIF) [file pone.0148965.s003.tif]
