# Supplementary material for: Genome-Wide De Novo Prediction of Cis-Regulatory Binding Sites in Mycobacterium tuberculosis H37Rv
Source: PLoS One. 2016 Feb 17;11(2):e0148965. doi: 10.1371/journal.pone.0148965 (PMC4757040; doi:10.1371/journal.pone.0148965)
Supplement: S1 File — (DOC) [file pone.0148965.s004.doc]

**Supplementary Materials**

1. **Selection of target genomes**
2. **Prediction of CRBSs using eGLECLUBS**
3. **Bacterial growth conditions, production of recombinant protein Rv0081 and RNA extraction**
4. **Primers for RT-PCR**
5. **Selection of target genomes**

Since CRBSs are relatively conserved in closely related genomes, often in the same class or even in the same order, we firstly chose all 158 available genomes (*M.tb* H37Rv included) of the order *actinomycetales* on NCBI. Transcriptional regulatory similarities of these genomes are evaluated by TF orthologs distribution vectors. We identified all the orthologs of the 172 TFs of *M.tb* H37Rv predicted by DBD database in each of the other 157 genomes of members of *actinomycetales* using BLASTP (E-value cutoff 10^-20^). The ortholog distribution of the 172 TFs of *M.tb* H37Rv in each of the 157 genomes was represented by generating 157 bit vectors Gi (b1, b2, …, bj, …, bn,), where bj = 1, if the j-th TF of *M.tb* has an ortholog in the genome Gi, otherwise bj = 0. Then we constructed a neighbor-joining tree based on the Hamming distance between each pair of these 157 TF orthologs distribution vectors using MEGA5 , and selected a monophyletic group including the target genomes in this tree with too closely related genomes removed (Fig S1). The set of target genomes contains 35 genomes belonging to the suborder *corynebacterineae* and each of them has at least 50% TF orthologs in the *M.tb* H37Rv genome (Fig S2).


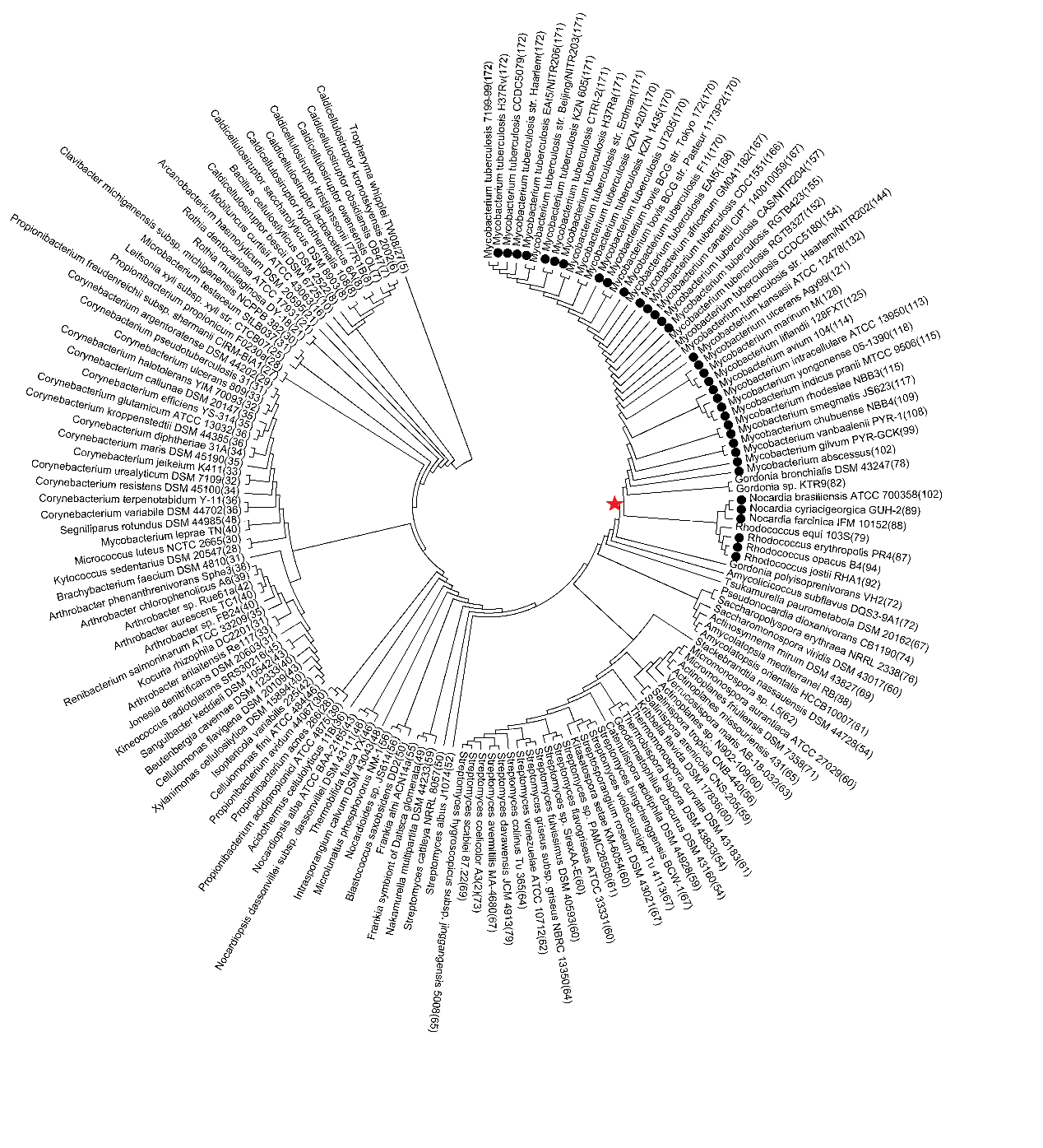


**Fig S1. Tree for the selection of reference genomes for the target genome *M.tb* H37Rv.** We construct this tree based on the Hamming distance between the TF distribution vectors of each pair of genomes. The number in each brace is the number of TFs shared with *M.tb* H37Rv. The 35 genomes labeled with a solid dot are selected as the reference genomes that belong to the monophyletic clades rooted at the star. Some high similar genomes in the clades are not included to avoid overrepresentation in reference genomes. Each reference genome has at least 50% TF orthologs sharing with the *M.tb* H37Rv genome.

**Fig S2. Tree for the 35 target genomes.** We construct this tree based on the Hamming distance between the TF distribution vectors of each pair of genomes. Phylogenetic tree was constructed using the Neighbor-Joining method. The number in each brace is the number of TF orthologs sharing with *M.tb* H37Rv.

1. **Prediction of CRBSs using eGLECLUBS**
2. **Identification of orthologous relationships between operons.**

First, we used the bi-directional best hits (BDBH) method to identify the orthologous relationships between any two genes from different genomes (E-value 10^-20^). Second, we identified the orthologous relationships between any two operons. If an operon contains >50% orthologous genes in the other operon, then the two operons were considered to be orthologous pairs. For each operon, we put all its orthologous pairs and itself in an operon set. Third, we connected any two operon sets if the larger one contains >70% of operons in the smaller one to construct a graph.

1. **Prediction of clusters of operons with orthologous relationships (COOR).**

We used markov cluster algorithm (MCL) to cut the graph above into clusters of operons with orthologous relationships (COOR). We then extracted up to 1000 bases upstream inter-operonic region of each operon in each COOR. COORs less than three orthologous sequences were discarded. A total of 11542 COORs were generated.

1. **Prediction of motifs for each COOR.**

Five motif-finding tools MEME , BioProspector , CUBIC , MDscan , and MotifSampler , which are complementary to each other , were used to identify motifs to each COOR. Different motif lengths from 8 to 22 bp were tested with all these programs and found that the motif length 16 bp performed best in recovering the known CRBSs . Therefore, 16 bp was used as the fixed motif length for all the five motif-finding tools.

Different combinations of the number of output motifs for each tool were compared as previously described . We selected a total of 40 motifs from the outputs of the five tools for each COOR, which included the top 15 of MEME, the top 10 of BioProspector, and the top five of CUBIC, MDscan, and MotifSampler, respectively. Some COORs contain too few orthologous sequences to generate enough motifs. In fact, a total of 444209 instead of 461680 in theory (11542×40) motifs were predicted and used as input motifs for the construction of motif similarity graph.

1. **Construction of motif similarity graphs.**

First, we constructed motif similarity graphs G1 and G2 using predicted motifs as the nodes. For any two nodes, if the similarity score is greater than a cut-off value, then the two nodes were connected by an edge. The similarity score for each pair of input motifs was computed using the motif similarity metric that Zhang *et al*. defined previously . According to eGLECLUBS , G1 must include the majority of input motifs and G2 must be efficiently clustered by the MCL algorithm. Thus, we select the cut-off α=0.27 for the construction of G1 that is large enough to include the vast majority of input motifs (99.99%) and the cut-off β=0.4 for construction of G2 that has a low enough density and still keeps more than 90% of the input motifs ( see Table 1 ) .

Table 1. Motif similarity graphs with different similarity score cutoffs.

| Similarity score cutoff | Nodes | Percentage | Edges | Density |
| --- | --- | --- | --- | --- |
| 0.25 | 444209 | 1 | 297637659 | 3.02E-03 |
| 0.26 | 444209 | 1 | 242549390 | 2.46E-03 |
| 0.27 | 444208 | 0.9999 | 196844887 | 2.00E-03 |
| 0.28 | 444204 | 0.9999 | 159140829 | 1.61E-03 |
| 0.3 | 443969 | 0.9995 | 102949450 | 1.04E-03 |
| 0.32 | 443086 | 0.9975 | 66001481 | 6.72E-04 |
| 0.34 | 440127 | 0.9908 | 42200639 | 4.36E-04 |
| 0.36 | 433157 | 0.9751 | 27128409 | 2.89E-04 |
| 0.38 | 419879 | 0.9452 | 17763790 | 2.02E-04 |
| 0.4 | 400515 | 0.9016 | 12002380 | 1.50E-04 |
| 0.41 | 388560 | 0.8747 | 10033504 | 1.33E-04 |
| 0.45 | 334943 | 0.7540 | 5510426 | 9.82E-05 |

We then construct and cluster the motif similarity graph G3, the quasi-clique-based motif similarity graph G4, the extended sequence-based motif similarity graph G5 by following the steps of eGLECLUBS exactly . G5 was clustered into a set of clusters using MCL, and each cluster contains binding sites for all 35 target genomes. Binding sites from the *M.tb* H37Rv genome in the clusters were extracted to form the H37Rv-specific clusters.

1. **Refine and rank the clusters of *M.tb* H37Rv.**

We followed the steps in eGLECLUBS to refine the H37Rv-specific clusters, which include predicting renewed motifs, clustering the renewed motifs, merging and extending sequences in each cluster, refining clusters using MEME again . A detailed description of these steps and the Perl scripts are available at the website <http://gleclubs.uncc.edu/pbs/>. After that, we ranked the clusters according to the similarity scores of clusters. The similarity score of a cluster was defined previously to evaluate the likelihood that a cluster contains a true motif . Each top-ranked cluster is expected to be a putative *cis*-regulatory binding motif of *M.tb* H37Rv.

1. **Bacterial growth conditions, production of recombinant protein Rv0081 and RNA extraction**

**growth conditions.** *M.tb* H37Rv strain was grown in liquid Middle-brook 7H9 medium and cultured at 37^o^C without extra oxygen until the exponential phase of growth reached standard conditions. The cultures were then subjected to anaerobiosis as described by Wayne and Hayes . The control group was cultured at 37^o^C under normal conditions and harvested at the same time.

**Plasmid construction.** *E. coli* DH5ɑ and *E. coli* BL21 (DE3) were used as host strains for plasmid construction and expression respectively. Primers used for amplification of nucleotide sequence of Rv0081 to construct plasmid pET28b-Rv0081 were the following: upper primer ATGGAGTCCGAACCGCTGT; lower primer GCGGGTGGTTCGGCTAT. Rv0081 was cloned into expression vector pET28b between the *Nhe*I and *Hind*III restriction sites. Same [strategy](javascript:void(0);) was used in constructing the *E. coli-Mycobacterium* shuttle vector pMV261-Rv0081. Primers pMV261-F (CCGGAATTCGTGGAGTCCGAACCG) and pMV261-R (CCCAAGCTTCGTGGCCGAGCCGCCGG) were used to amplify Rv0081 and then cloned into the constitutive expression plasmid pMV261 between the *Eco*RI and *Hind*III restriction sites. Vector pMV261-Rv0081 was cloned into H37Rv by electrotransformation.

**Production and purification of recombinant protein Rv0081 in *E. coli.*** *E. coli* BL21 (DE3) strain transformed with plasmid pET28b was grown overnight on selective LB agar medium containing kanamycin, suspended in LB selective broth, and grown to mid-exponential phase. 0.1mM IPTG were added to induce protein overproduction for five hours. Induced cells were suspended in lysis buffer (20mM Tris-HCl, 200mM NaCl, 5mM imidazole, pH= 8.0), passed 3 times through low temperature ultra-high pressure continuous flow cell disrupter (JNBIO), and centrifuged at 10,000×g for 30 min. Cellular supernatants were passed over a HisTrap^TM^ HP column (GE), and collected fractions were pooled. Purified protein fractions were [ultrafiltrated](app:ds:ultrafiltration) against dialysis buffer (20 mM Tris-HCl, 150mM NaCl, 20% glycerol, pH=8.0) with Amicon Ultra-15 (MWCO 10K, Millipore). Purified proteins were stored at -80^o^C in dialysis buffer after quick froze by liquid nitrogen.

**RNA extraction.** Appropriate H37Rv strains were collected at exponential phage (after 14 days in this study) and centrifuged at 4,500×g for 5 min at room temperature, and frozen on dry ice. The frozen cell pellets were mixed with glass beads d in 1ml of TRIzol reagent (CW-bio) and disrupted in an oscillator (MP, Fastprep-24). RNA extraction was performed as previously described .

1. **Primers for RT-PCR**

Real-time PCR were done in technical triplicates. CT values were normalized against *sigA* expression, and fold change was calculated by the 2^− ΔΔCT^ method. Primers for RT-PCR are described in table 2.

Table 2. Primers for RT-PCR

| name | sequence |
| --- | --- |
| Rv0002-F | TTT AGT TTC TGG CCG ATT GTT |
| Rv0002-R | ATG CGA CCC GGT TAC CTT C |
| Rv0003-F | TGT TGG GCC TAA CGG TTA TGG |
| Rv0003-R | GGC AAA TCG GCG CTA A |
| Rv0004-F | AGA AAC GCG GCT GGT CGG T |
| Rv0004-R | GCG TGC ATG TTC GGC GAT CTG |
| Rv0505c-F | GCC TTC TTC GAC GTG GAC AAC |
| Rv0505c-R | GCG GTA GGT GAA GTA GTG G |
| Rv1057-F | GGT CAA GAT TCC GGT GCA G |
| Rv1057-R | CGG TGC CGT TGT TGG TCA CTA |
| Rv1503c-F | CGC TTC CTT CGC AAC GAG |
| Rv1503c-R | CCA TAG AAA GGC AGC GAC TAA |
| Rv1504c-F | TCA TAC TGC CGT CAT ACA CCT |
| Rv1504c-R | GGC GTC TAC GAT GCG AGT TTC |
| Rv2145c-F | AAC GCA AGC ACT CCG AGA TCA |
| Rv2145c-R | TGG TGC GGT ACT CAC GTT C |
| Rv2329c-F | GCG TTC CTG CGG ATG CCC TAC |
| Rv2329c-R | GGC CGG AAT TAG CAA CAC GAT |
| Rv2699c-F | ACT GTC GGT GCG CGT CGT A |
| Rv2699c-R | GCA GAT CAT CAC GCC GTT CTT |
| Rv2788c-F | GGC TGT CGG GTT ACG GA |
| Rv2788c-R | CTG CAG GGT GCG TCG AAT GTG |
| Rv3619c-F | TGT TGA CCG CGA GTG AC |
| Rv3619c-R | TTG GCC TGC TCG TAG ATC ACC |

References

1. Tamura K, Peterson D, Peterson N, Stecher G, Nei M, et al. (2011) MEGA5: molecular evolutionary genetics analysis using maximum likelihood, evolutionary distance, and maximum parsimony methods. Mol Biol Evol 28: 2731-2739.

2. Mushegian AR, Koonin EV (1996) A minimal gene set for cellular life derived by comparison of complete bacterial genomes. Proc Natl Acad Sci U S A 93: 10268-10273.

3. Enright AJ, Van Dongen S, Ouzounis CA (2002) An efficient algorithm for large-scale detection of protein families. Nucleic Acids Res 30: 1575-1584.

4. Bailey TL, Elkan C (1994) Fitting a mixture model by expectation maximization to discover motifs in biopolymers. Proc Int Conf Intell Syst Mol Biol 2: 28-36.

5. Liu X, Brutlag DL, Liu JS (2001) BioProspector: discovering conserved DNA motifs in upstream regulatory regions of co-expressed genes. Pac Symp Biocomput: 127-138.

6. Olman V, Xu D, Xu Y (2003) CUBIC: identification of regulatory binding sites through data clustering. J Bioinform Comput Biol 1: 21-40.

7. Liu XS, Brutlag DL, Liu JS (2002) An algorithm for finding protein-DNA binding sites with applications to chromatin-immunoprecipitation microarray experiments. Nat Biotechnol 20: 835-839.

8. Thijs G, Lescot M, Marchal K, Rombauts S, De Moor B, et al. (2001) A higher-order background model improves the detection of promoter regulatory elements by Gibbs sampling. Bioinformatics 17: 1113-1122.

9. Hu J, Li B, Kihara D (2005) Limitations and potentials of current motif discovery algorithms. Nucleic Acids Res 33: 4899-4913.

10. Zhang S, Xu M, Li S, Su Z (2009) Genome-wide de novo prediction of cis-regulatory binding sites in prokaryotes. Nucleic Acids Res 37: e72.

11. Zhang S, Li S, Pham PT, Su Z (2010) Simultaneous prediction of transcription factor binding sites in a group of prokaryotic genomes. BMC Bioinformatics 11: 397.

12. Wayne LG, Hayes LG (1996) An in vitro model for sequential study of shiftdown of Mycobacterium tuberculosis through two stages of nonreplicating persistence. Infect Immun 64: 2062-2069.

13. Maciag A, Dainese E, Rodriguez GM, Milano A, Provvedi R, et al. (2007) Global analysis of the Mycobacterium tuberculosis Zur (FurB) regulon. J Bacteriol 189: 730-740.
